# Myxoma Virus and the Leporipoxviruses: An Evolutionary Paradigm

**DOI:** 10.3390/v7031020

**Published:** 2015-03-06

**Authors:** Peter J. Kerr, June Liu, Isabella Cattadori, Elodie Ghedin, Andrew F. Read, Edward C. Holmes

**Affiliations:** 1 CSIRO Biosecurity Flagship, Black Mountain Laboratories, Clunies Ross Street, Acton, ACT 2601, Australia; E-Mail: june.liu@csiro.au; 2 Center for Infectious Disease Dynamics, Department of Biology, The Pennsylvania State University, University Park, PA 16802, USA; E-Mails: imc3@psu.edu (I.C.); a.read@psu.edu (A.F.R.); 3 Center for Genomics and Systems Biology, Department of Biology and Global Institute of Public Health, New York University, New York, NY 10003, USA; E-Mail: elodie.ghedin@nyu.edu; 4 Marie Bashir Institute for Infectious Diseases and Biosecurity, Charles Perkins Centre, School of Biological Sciences, and Sydney Medical School, The University of Sydney, Sydney, NSW 2006, Australia; E-Mail: edward.holmes@sydney.edu.au

**Keywords:** myxoma virus, leporipoxvirus, poxvirus, myxomatosis, rabbit, coevolution

## Abstract

Myxoma virus (MYXV) is the type species of the *Leporipoxviruses*, a genus of *Chordopoxvirinae*, double stranded DNA viruses, whose members infect leporids and squirrels, inducing cutaneous fibromas from which virus is mechanically transmitted by biting arthropods. However, in the European rabbit (*Oryctolagus cuniculus*), MYXV causes the lethal disease myxomatosis. The release of MYXV as a biological control for the wild European rabbit population in Australia, initiated one of the great experiments in evolution. The subsequent coevolution of MYXV and rabbits is a classic example of natural selection acting on virulence as a pathogen adapts to a novel host species. Slightly attenuated mutants of the progenitor virus were more readily transmitted by the mosquito vector because the infected rabbit survived longer, while highly attenuated viruses could be controlled by the rabbit immune response. As a consequence, moderately attenuated viruses came to dominate. This evolution of the virus was accompanied by selection for genetic resistance in the wild rabbit population, which may have created an ongoing co-evolutionary dynamic between resistance and virulence for efficient transmission. This natural experiment was repeated on a continental scale with the release of a separate strain of MYXV in France and its subsequent spread throughout Europe. The selection of attenuated strains of virus and resistant rabbits mirrored the experience in Australia in a very different environment, albeit with somewhat different rates. Genome sequencing of the progenitor virus and the early radiation, as well as those from the 1990s in Australia and Europe, has shown that although MYXV evolved at high rates there was no conserved route to attenuation or back to virulence. In contrast, it seems that these relatively large viral genomes have the flexibility for multiple pathways that converge on a similar phenotype.

## 1. Introduction

The introduction of myxoma virus (MYXV) into the European rabbit (*Oryctolagus cuniculus*) population of Australia is the classic example of host-pathogen coevolution following a species jump. In its natural host, *Sylvilagus brasiliensis*, the South American tapeti or forest rabbit, infection with MYXV causes an innocuous cutaneous fibroma, which persists for some weeks. Virus is passively transmitted from the fibroma on the mouthparts of biting arthropods. This benign host-pathogen coexistence has been presented as a text book example of an evolutionary climax in which the pathogen causes little harm to the host and both host and pathogen survive [1]. The species jump to the European rabbit in which MYXV causes the lethal, generalized disease myxomatosis and the subsequent coevolution of the virus and its new host, with selection for more attenuated strains of virus and increased resistance of the host, could be seen as the first steps towards this equilibrium. However, as May and Anderson (1983) [2] have pointed out, this theoretical climax state can only arise if transmission and duration of infectivity are independent of virulence. If this is not the case, then the potential coevolutionary pathways followed will depend on the nexus between transmission and virulence of the pathogen and the costs of developing resistance in the host. The ongoing coevolution of MYXV and the European rabbit beautifully illustrates the exploration of these pathways and the ensuing evolutionary arms race between host and parasite.

Between March and November 1950, MYXV was experimentally inoculated into wild European rabbits at five field study sites in the Murray Valley of south eastern Australia. The aim was to re-evaluate the virus as a biological control for rabbits. As expected, MYXV spread poorly through the local rabbit populations and quickly died out, or so it appeared, confirming earlier studies in Australia and Europe that MYXV had limited potential as a biological control agent [3,4,5,6,7]. The re-emergence of MYXV in December 1950 and its spread over much of SE Australia was unexpected and unprecedented in scale and lethality [3,8]. Thus, inadvertently, began one of the great experiments in natural selection, conducted on a continental scale and, remarkably, repeated on the same scale, in a different environment, with the deliberate release of a second strain of MYXV in France, which spread through Europe. On both continents, there was selection for attenuated strains of virus, which, because the infected rabbit survived for longer, were more likely to be transmitted by insect vectors, and may have facilitated selection for genetically resistant rabbits. What made these such important and unique natural experiments was that the original virus strains released were available for comparison with subsequent field strains, and the wild European rabbit is the same species as the domestic/laboratory rabbit, with similar outcomes following infection. This meant that detailed evolutionary studies could be conducted on both the virus and its new host using laboratory rabbits as unselected controls and, given the short generation time of the rabbit and the relatively rapid changes in the virus, natural evolution could be measured in real time.

## 2. MYXV and the Leporipoxviruses

MYXV is the type species of the *Leporipoxvirus* genus (family: *Poxviridae*; subfamily *Chordopoxvirinae*). The virus naturally infects the South American tapeti*,* causing a cutaneous fibroma at the inoculation site. However, in the European rabbit, which is exotic to the Americas, MYXV causes myxomatosis, a generalized, lethal disease characterized by swollen head, eyelids and ears, raised cutaneous lesions over the body, ears and legs, ano-genital oedema, blepharoconjunctivitis and mucopurulent ocular and nasal discharge. This disease was first described following an outbreak in laboratory rabbits at the Institute of Hygiene in Montevideo, Uruguay in 1896 [9].

**Table 1:** The *leporipoxviruses*.

Virus	Natural Host	Disease Caused in Natural Host	Geographic Distribution of Natural Host
Myxoma virus	*Sylvilagus brasiliensis* (tapeti)	Cutaneous fibroma	South & Central America
Californian Myxoma virus	*Sylvilagus bachmani* (brush rabbit)	Cutaneous fibroma	West coast USA & Mexico
Rabbit (Shope) fibroma virus	*Sylvilagus floridanus* (eastern cottontail)	Cutaneous fibroma	Eastern & central North America to Central and South America
Squirrel fibroma virus	*Sciurus carolinensis* (eastern gray squirrel) ^1^	Cutaneous epitheliofibroma; may be generalized and involve internal organs	Eastern North America; introduced in Europe and Britain
Hare fibroma virus	*Lepus europaeus* (European brown hare) ^2^	Cutaneous fibroma	Europe but widely introduced to other countries
Western squirrel fibroma virus	*Sciurus griseus griseus* (western gray squirrel)	Cutaneous thickening	West coast of North America

^1^ Woodchucks (*Marmota monax*) are also susceptible to experimental infection [10,11]; ^2^ *Lepus californicus* (Californian jack rabbit) was susceptible experimentally [12]; poxvirus lesions have also been described in *Lepus capensis* (Cape hare) in Kenya [13] but no virus characterization was done.

Leporipoxviruses closely related to MYXV are present in *Sylvilagus floridanus* (eastern cottontail), which is native to the eastern parts of North America and extending south into Central America and in *S. bachmani* (brush rabbit) found on the west coast of North American and into Mexico. However, infections with these viruses cause very different outcomes in European rabbits. Rabbit fibroma virus (RFV), found in *S. floridanus*, induces a cutaneous fibroma at the inoculation site in immunocompetent European rabbits and is used as a heterologous vaccine against myxomatosis [14,15]. In contrast, Californian strains of MYXV (Cal MYXV) from *S. bachmani* have extreme virulence for European rabbits, often killing the rabbit before the classic signs of myxomatosis can develop [16,17]. Other leporipoxviruses that serologically cross-react with MYXV [18,19]; have been identified in squirrels in the Americas and hares (*Lepus europaeus*) in Europe (Table 1).

### 2.1. The Biology of the Leporipoxviruses in Their Natural Hosts

#### 2.1.1. Myxoma Virus (MYXV)

Fibromas induced by MYXV in *S.brasiliensis* persist for some weeks followed by regression. Occasionally a more generalized disease may occur [8,20]. Virus is passively transmitted by adhering to the mouthparts of biting arthropods such as mosquitoes as they probe through the fibroma for a blood meal. Natural infection will almost certainly be via flea or mosquito. However, under experimental conditions, *S. brasiliensis* could also be infected by conjunctival inoculation or by direct contact with a European rabbit with myxomatosis [20]. The duration of immunity to reinfection is not known [8,20]. The distribution of MYXV in the Americas originally followed the distribution of the tapeti through north eastern Argentina, Brazil and into Central America [18], but the virus has subsequently established in Chile and southern Argentina, where *S. brasiliensis* is not present, following its introduction to control the spread of European rabbits [18,21,22].

#### 2.1.2. Californian MYXV (Cal MYXV)

Cal MYXV induces a cutaneous fibroma in *S. bachmani* (brush rabbit) from which it can be transmitted by mosquitoes. In brush rabbits infected experimentally, most of the fibromas scabbed within four weeks, and thus became unsuitable for mosquito transmission, but some persisted for much longer providing a source of infection for 2–3 months [23,24]. It was suggested that MYXV was introduced to farmed European rabbits in California in 1928 via a shipment of rabbits from Mexico, and outbreaks were recorded in the early 1930s [8,25,26]. However, a sylvatic cycle in the brush rabbit population and mosquitoes was subsequently demonstrated [23,27,28]. Myxomatosis has been reported in farmed European rabbits from Oregon to the Baja peninsula of Mexico [29,30]. This wide distribution, which coincides with that of the brush rabbit, suggests that the virus has probably been present in this locality for a long time, but raises interesting questions about its ultimate origins since there is currently no geographic overlap between *S. bachmani*, which has a limited distribution along the west coast of North America, and *S. brasiliensis* or *S. floridanus*.

#### 2.1.3. Rabbit Fibroma Virus (RFV)

RFV, also known as Shope fibroma virus, is the best studied of the leporipoxviruses in its natural host and provides a model for their biology. RFV normally infects *S. floridanus* (eastern cottontails) [31,32]; and virus is readily mechanically transmitted by mosquitoes, fleas and other biting arthropods [33,34,35]. Following inoculation, RFV replicates at the site of infection, which is usually on the feet or other thinly haired areas that are attractive to mosquitoes, causing a cutaneous fibroma, 1–2 cm in diameter, with hyperplasia and hypertrophy of the overlying epidermal cells. This fibroma can persist for some months in the face of an ongoing immune response before being cleared, although infected cottontails are refractory to further infection from around six days; recovered cottontails are immune to reinfection [36]. Infectivity of the fibroma for mosquitoes is associated with high titres of virus in the epidermis, which occurs quite late in the infection, around 30–35 days, and is maintained until the fibroma scabs. Infection of neonatal kittens can lead to uncontrolled growth of the fibroma or generalized disease [37]. A fascinating adaptation by RFV is persistence of infective fibromas in cottontails infected as young kittens, thereby allowing the virus to overwinter in the absence of mosquitoes and susceptible kittens; experimentally, infectivity was maintained as long as 10 months, although even cottontails infected as adults can retain infective fibromas for up to seven months [35]. RFV has been reported from Ontario in Canada to Texas in the USA suggesting that the virus follows the broad distribution of the eastern cottontail [38,39]. *S. floridanus* has also been introduced into Europe for hunting but whether RFV was also inadvertently introduced is not known [40,41].

#### 2.1.4. Hare Fibroma Virus

Hare fibroma virus is the only leporipoxvirus naturally found outside the Americas. It induces relatively large (1–3 cm diameter), protuberant cutaneous fibromas in European hares (*Lepus europaeus*) [42]. The origins and epizootiology of the virus are obscure but outbreaks in Europe were described as early as 1909, well before the introduction of myxomatosis to the continent, which can occasionally cause infections of European hares [8,43,44]. It is presumed that, like the other leporipoxviruses, hare fibroma virus is passively transmitted by biting arthropods. Experimentally, the virus induced large fibromas in the American hare *Lepus californicus* (black tailed jack rabbit) [12] and small fibromas in European rabbits [18] but it is not known if these can support transmission. A similar fibroma, containing poxvirus particles, has been reported in Cape hares (*Lepus capensis*) from Kenya [13], but virus could not be propagated in a European rabbit and no serological characterization was done.

#### 2.1.5. Squirrel Fibroma Virus

Squirrel fibroma virus induces cutaneous fibromas and proliferative epidermal lesions in eastern gray squirrels (*Sciurus carolinensis*) in North America. Generalized disease can occur in suckling squirrels with proliferative lesions over the body and in the lungs, liver, lymph nodes and kidney [10,11,45,46]. Infection was readily transmitted from infected young squirrels to juveniles by mosquitoes, but adult squirrels were difficult to infect and virus titres in the resulting fibromas were not sufficient for mosquito transmission [10]. However, fibromas and generalized disease have been reported in naturally infected adult squirrels [10,47,48]. Given the apparent difficulty in transmission it is not clear how the virus is maintained in the population over the winter period between breeding cycles. It is also possible that the squirrel is not the definitive host or that a relatively recent species jump has occurred. Juvenile woodchucks (*Marmoto monax*) are also susceptible to infection and can transmit virus via mosquito feeding [10,11]. Fibromas have also been reported in a fox squirrel (*Sciurus niger*) and porcupines (*Erethizon dorsatum*) from the eastern USA [49], but whether these were due to squirrel fibroma virus is not known.

A putative leporipoxvirus has been described from skin lesions on western gray squirrels (*Sciurus griseus griseus*) in California. Classification as a leporipoxvirus was based on serological cross-reaction with Californian MYXV, although it did not cross-react with South American MYXV and electron microscopy showed particles with surface striations that were not typical of other leporipoxviruses [50].

### 2.2. Species Specificity of Leporipoxviruses

Divergence from a putative common ancestral leporipoxvirus likely occurred either by shifts into new species and subsequent adaptation or ongoing host speciation and geographic separation. The known leporipoxviruses appear to have narrow host ranges with only closely related species susceptible to infection [8,51,52]. Moreover, transmission is generally even more restricted. This can be seen from experiments with the natural hosts of MYXV, Cal MYXV and RFV. MYXV replicated in *S. bachmani* forming cutaneous tumours but did not reach sufficient titres to be transmitted by mosquitoes [53,54]. Cal MYXV transferred by mosquito did not cause visible infection in *S. brasiliensis*, and although visible fibromas were formed in *S. floridanus* no transmission could be achieved from this species due to low virus titres in the fibromas [12]. RFV did not induce a lesion in *S. bachmani* (cited in [8]). However, occasional mosquito transmission of MYXV from experimentally infected *S. floridanus* has been reported [54].

Interestingly, while RFV can replicate to high titres in the fibroma on European rabbits, it could only rarely be transmitted by mosquitoes, probably because the immune response controls the fibroma before high titres of virus are reached in the overlying epidermis, which appears critical for transmission by mosquitoes [55,56]. Despite the extensive use of RFV as a vaccine in farmed and wild European rabbits in France [40,41,57], there is no evidence of a natural transmission cycle.

Two species of North American cottontails, *S. audubonii* and *S. nuttalli*, were susceptible to experimental infection with MYXV and efficient sources of virus for mosquito transmission [54]. The distribution of *S. audubonii* overlaps with *S. bachmani* but this species does not support transmission of Cal MYXV with virus infection inducing a rapid and intense T cell response at the inoculation site [12,58]. In addition, European hares and mountain hares (*Lepus timidus*) can occasionally be infected, naturally or experimentally, with MYXV but are generally refractory [8,43,44,52].

The establishment of MYXV in European rabbits demonstrates that if a susceptible host is available then species jumps can be successful. However, on many occasions such infections must simply burn out without establishing an ongoing transmission cycle because the host does not support efficient transmission, the population size is too limited, the infection is too virulent or vectors are inefficient. This seems to have been the fate of the early attempts to introduce MYXV in Europe and Australia and of infections that occurred in domestic and laboratory European rabbits in South America [6,7,8].

### 2.3. Genome Sequences of MYXV and Related Leporipoxviruses

The Lausanne (Lu) strain of MYXV (Brazil/Campinas 1949) has a genome of 161,777 bp of double stranded DNA with closed single strand hairpin termini. It encodes 158 unique open reading frames (ORFs), 12 of which are duplicated in the 11,577 bp terminal inverted repeats (TIRs) [59,60]. By convention, genes are prefixed by M and numbered from the left hand (LH) end of the genome with the direction of transcription shown as left (L) or right (R). Genes within the TIRs are labelled as L/R. This system has also been used for RFV (but with genes prefixed by S) and Cal MYXV [61,62].

Genes encoding proteins involved in replication and structure are relatively conserved with other poxviruses and tend to be in the central part of the genome [59,63], while genes towards the termini tend to encode host-range and virulence factors. There are at least 42 genes with demonstrated or potential host-range or immunomodulatory effects many of which have been demonstrated to have roles in virulence in European rabbits (Table 2) [64]. Although these genes have obviously undergone selection in the natural host of MYXV, all our knowledge of the function of their encoded proteins *in vivo* comes from studies in European rabbits.

The Kasza strain of RFV has been completely sequenced [61]. The genome of 159,857 bp is slightly smaller than Lu due to loss of seven genes (Table 3), but there are no novel genes in the 151 unique ORFs. The TIRs are slightly longer at 12,397 bp, with the TIR boundary within the *S009* gene, which means most of this ORF and all of the *S008.2* ORF, which is the equivalent of the *M156R* gene in Lu, are duplicated, but leaving the RH copy of *S009* as a truncated pseudogene. Apart from the expansion of the TIRs, the gene order is conserved between Lu and RFV. Gene loss appears to have proceeded by fragmentation and gradual loss of the ORFs. At least six genes implicated in virulence or with potential immunomodulatory functions in Lu are missing from RFV, which may explain why this virus is unable to disseminate and replicate at distal sites in immunocompetent European rabbits. Immune suppressed or very young European rabbits are however susceptible to more generalized disease following inoculation of RFV [38,65,66,67].

The MSW strain of Cal MYXV has a genome of 164,600 bp. This increase in size, compared to the other sequenced leporipoxviruses, is predominantly due to an expansion of the TIRs to 15,464 bp with the *M156R*, *M154R*, *M153R*, *M152R*, *M151R* ORFs and part of the *M150R* ORF from the RH end of the genome duplicated and inserted in the LH TIR with deletion of a large part of the *M009L* ORF [62]. MSW is more than 10% diverged from Lu at the nucleotide level. However, comparison of the protein sequences for Lu, RFV and MSW demonstrates that MSW is clearly more closely related to Lu than it is to RFV and, despite the larger genome and extreme virulence for European rabbits, there are no novel genes in MSW. Once allowance is made for the expansion of the TIRs, the gene order is identical to Lu (and RFV).

**Table 2:** Genes in myxoma virus encoding demonstrated or potential immunomodulatory or host-range proteins.

Gene	Protein Function (no. of Amino Acids; Transcription Time: E [Early], L [Late])	Reference	Effect on Virulence of Gene Disruption
*M001L/R*	chemokine binding (260; E)	[68]	Generalized myxomatosis; 1/6 survived
*M002L/R*	TNF binding; antiapoptosis (326; E)	[69,70,71,72]	Moderate to severe myxomatosis; 5/8 animals survived
*M003.1L/R*	VACV ^1^ B15 orthologue; Bcl-2 fold (151; E)	[73]	Not determined
*M004L/R*	RDEL motif; antiapoptosis (237; E)	[74,75]	Small rapidly resolved primary lesions; 1/8 rabbits had a secondary; all animals recovered
*M005L/R*	Antiapoptosis; E3 Ub ligase(483; E)	[76]	Primary lesion only; rapid resolution; no signs of clinical myxomatosis
*M006L/R*	BTB/kelch domains; putative E3 Ub ligase (509; E)	[77]	Not determined
*M007L/R*	Secreted IFN- γ binding protein; chemokine binding (263; E)	[78,79]	12/13 rabbits mild to moderate disease; lymphocyte infiltration
*M008L/R*	BTB/kelch domains; putative E3 Ub ligase (515; E)	[77]	Not determined
*M008.1L/R*	Serp 1; secreted serine proteinase inhibitor (369; L)	[80]	Moderate to severe generalized myxomatosis; 5/8 rabbits recovered from infection; enhanced inflammatory response
*M009L*	BTB/kelch domains; putative E3 Ub ligase (509; E)	[77]	Not determined
*M010L*	Epidermal growth factor homologue (85; E)	[81]	Generalized myxomatosis; 75% of animals recovered
*M011L*	Antiapoptotic factor (166; E)	[81,82]	All rabbits survived; large protuberant demarcated primary; large secondaries; mild conjunctivitis/rhinitis
*M013L*	Pyrin domain inflammasome (126; E)	[83]	Mild clinical signs rapidly resolved; small secondaries; no mortality; rapid inflammatory response
*M014L*	BTB/kelch domains; putative E3 Ub ligase (517; E)	[77]	Not determined
*M029L*	Type I interferon resistance/PKR inhibition; RNA helicase A binding (115; E)	[84,85]	Abortive infection
*M036L*	VACV O1 orthologue; ERK1/2 signal enhancement (680; E)	[86]	Not determined
*M062R*	Host range (158;E/L )	[87]	abortive infection in rabbits and rabbit cells
*M063R*	Host range (215; E)	[88]	No virus replication in rabbits and rabbit cells
*M064R*	Virion component (203; E/L)	[89]	Slower progression of disease but lethal in rabbits
*M104L*	potential immunomodulatory (53; L)	[59]	Not determined
*M121R*	NK cell receptor homologue (176; E)	[59]	Not determined
*M122R*	NK cell receptor homologue (172; L)	[59]	Not determined
*M128L*	CD47 homologue; macrophage inhibition (281; L)	[90]	Mild generalized disease; rapid resolution; no deaths
*M130R*	Unknown function; localized to ER/Golgi, glycosylated but not secreted (122; L)	[91]	Generalized myxomatosis but no deaths
*M131R*	Superoxide dismutase inhibition (163; L)	[92,93,94]	All animals euthanized days 10-11; RFV is attenuated
*M135R*	Immunomodulatory (178; E)	[95]	Mild disease with little generalization; all survived
*M136R*	Homology to VACV A52; Bcl-2 fold (179; L?)	[59,73,96]	Not determined
*M138L*	Sialyltransferase (290; E)	[97]	Severe fatal myxomatosis; survival time prolonged
*M139R*	Homology to VACV A52; Bcl-2 fold (188; E)	[73]	Not determined
*M140R*	BTB/kelch domains; putative E3 Ub ligase (553; E?)	[59,77]	Not determined
*M141R*	OX-2 homologue (218; E)	[98]	Mild generalized disease, rapid resolution, all survived; increased macrophage and T cell activation
*M143R*	RING-E3 Ub ligase; possible apoptosis regulator (234; L)	[99,100]	Not determined
*M146R*	VACV N1 orthologue;TLR signal inhibition; Bcl-2 fold antiapoptosis (108; E?)	[59,73,96]	Not determined
*M148R*	Ankyrin repeat; putative E3 Ub ligase (675; L)	[77,101]	Moderate generalized; 2/5 rabbits euthanized at 21 days; mononuclear inflammatory response
*M149R*	Ankyrin repeat; putative E3 Ub ligase (490; E/L?)	[77,101]	Moderate generalized with delayed secondaries; 5/5 rabbits survived
*M150R*	NF-κB inhibition; E3 Ub ligase (494; E)	[77,102,103]	Rapid inflammatory response at primary site; few small secondaries; no respiratory disease; 12/12 recovered by d21
*M151R*	Serp 2 (333; E)	[104]	Primary lesion but few or no secondary lesions; 7/10 recovered
*M152R*	Serp 3 (266; L)	[105]	4/10 infected rabbits recovered; 6/10 euthanized because of respiratory disease; no secondary lesions
*M153R*	MHC downreg; E3 Ub ligase (206; E)	[106,107,108]	Generalized myxomatosis; 4/12 rabbits euthanized day 14, the remainder recovered
*M154L*	Downregulation of NFκB? Vac M2 orthologue (214; E)	[59,109]	Not determined
*M156R*	interferon resistance; eIF2α homologue (102; L/E?)	[110]	Not determined

^1^ VACV—vaccinia virus.

**Table 3:** Genes missing or substantially modified in RFV compared to Lu.

Lu Gene	Function	RFV Compared to Lu [59,61]
*M000.5L/R*	Unknown	Missing from RFV
*M008.1L/R*	Secreted serine proteinase inhibitor (Serp 1)	Fragmented in RFV
*M023R*	Unknown	35 aa in RFV; 61 aa in MYXV
*M119L*	unknown	N-terminal truncation
*M129R*	Unknown	78 aa in RFV; 136 aa in MYXV
*M135R*	Immunomodulatory	Fragmented in RFV
*M136R*	Possible immunomodulatory	Fragmented in RFV
*M139R*	Possible immunomodulatory	Fragmented in RFV
*M150R*	NFκB signal inhibition	Fragmented in RFV
*M152R*	Serp 3	Fragmented in RFV
*M156R*	eIF2α homologue (IFN resistance)	Truncated N-terminus; duplicated in RFV

Surprisingly, given the extreme virulence of MSW for European rabbits [16,17], there are six ORF disruptions (Table 4) including the *M008.1L*/*R* and *M152L*/*R* genes, which have been implicated in virulence for European rabbits in Lu [80,105,111]. Unlike the missing and fragmented genes in RFV [61], the original ORFs can be readily discerned and largely aligned with the Lu orthologues, although all have multiple nonsense mutations, suggesting that they have been disrupted much more recently than occurred for RFV. The overlap of disrupted genes between RFV and MSW is also interesting with *M000.5L*/*R*, *M008.1L*/*R* and *M152R* lost in both viruses (although whether *M000.5L*/*R* is transcribed in MYXV is unknown).

Based on these three genomes, the South American Lu strain appears to have a full complement of genes suggesting that it resembles a putative ancestral leporipoxvirus, although from the RFV genome, Willer *et al*. (1999) [61] have proposed that an ancestral virus may have had longer TIRS. There are relatively few gaps in the MYXV genome where gene loss may have occurred and no obviously fragmented genes although, compared to other poxviruses such as vaccinia virus (VACV), there are multiple genes that may have been lost at some point in leporipoxvirus evolution. For example, some 27 genes present in VACV (Copenhagen strain) are absent in the leporipoxviruses, including genes for three proteins involved in nucleotide synthesis, thymidylate kinase; guanylate kinase; and the large subunit of ribonucleotide reductase (although the small subunit is present). In addition, the glutaredoxin 1 gene is absent and the A type inclusion body genes are not present [59,61].

In contrast to MYXV, both MSW and RFV have multiple gene disruptions suggesting that they may have undergone host switches and adaptation with loss of genes, perhaps to decrease virulence and in turn increase transmissibility. Alternatively, the genes that were lost may not be required for replication in the new host. This appears to have happened more recently in MSW than in RFV, based on the fragmentation and loss of the genes in RFV compared to multiple indels in MSW.

**Table 4:** Genes missing or substantially modified in MSW compared to Lu.

Lu Gene	Function	MSW Compared to Lu [62]
*M000.5L/R*	Unknown	ATG but no ORF in MSW
*M008.1L/R*	Secreted serine proteinase inhibitor (Serp 1)	Multiple stop codons in MSW
*M009L*	BTB/kelch domains; potential E3 Ub ligase	Loss of 845 nts and multiple indels in MSW
*M023R*	Unknown	Mutation of ATG and disruption of ORF in MSW
*M077L*	Assembly complex	23 aa extra at N-terminus in MSW
*M119L*	Unknown	N-terminal truncation in MSW
*M131R*	Superoxide dismutase homologue	Multiple stop codons in MSW
*M152R*	Serp 3	Multiple stop codons in MSW
*M156R*	eIF2α homologue (IFN resistance)	Truncated N-terminus; gene duplicated in MSW

## 3. Pathogenesis of MYXV in European Rabbits

In European rabbits, virulent MYXV inoculated intradermally replicates in MHC-II + cells at the dermal/epidermal interface and then spreads to the draining lymph node, where it is found initially in the cells of the subcapsular sinus and then in lymphocytes in the T cell zones of the node. From here, the virus disseminates to distal tissues carried in lymphocytes and possibly monocytes; there is little or no free virus in the bloodstream [112,113,114]. At the same time, the virus infects epidermal cells at the inoculation site inducing cellular hyperplasia and hypertrophy with development of a raised cutaneous primary lesion overlying a disrupted dermis and subdermis swollen with the mucoid material from which the virus derives its name. Virus titres in lymphoid tissues can be very high (>10^8^ pfu/g) with profound depletion of lymphocytes from lymph nodes and, depending on the strain, spleen. Very high titres of virus occur in secondary cutaneous lesions, the swollen eyelids and particularly the very swollen tissues at the base of the ears, which are probably important for insect transmission. Virus is also found in tissues such as lung and liver but generally at lower titres [112,113,115]. Virus shedding occurs from conjunctivae and nasal passages and from the eroded surfaces of cutaneous lesions. Secondary bacterial infection of the upper respiratory tract and conjunctivae with gram negative bacteria such as *Pasteurella multocida* and *Bordetella bronchiseptica* is common, but early studies reported that internal organs were usually free of bacteria [116]. Bacterial pneumonia is not uncommon in rabbits infected with more attenuated viruses with longer survival times [117,118].

The ability of the virus to replicate and disseminate in lymphocytes and establish and replicate to high titres at distal sites is critical for virulence in European rabbits (reviewed in [64,119]). It is likely that the key viral proteins that support replication and dissemination evolved in *Sylvilagus* species to suppress the local immune response sufficiently to allow virus to persist at the cutaneous site, but in European rabbits these proteins profoundly suppress the immune system, which results in fatal disease. The actual cause of death is obscure. Mims (1964) [120] could not identify any vital organ damage and other workers have speculated that upper respiratory tract occlusion is the cause of death [116], but this idea is contradicted by Hurst (1937) [115] and rabbits infected with Californian strains of virus often die with little or no upper respiratory tract obstruction [16,17]. Duclos *et al*. (1983) [117] suggest that pulmonary oedema is important and this has been observed with modern Australian isolates, although not with the virulent progenitor strain (unpublished data; [115]).

## 4. Myxoma Virus Evolution in Australia

### 4.1. The European Rabbit and Initial Trials with MYXV

The European rabbit likely evolved in the Iberian Peninsula and southern France from where it was distributed around Europe often as semi-domesticated stock, managed for meat, fur, and hunting, that later reverted to the wild. The current domestic breeds were developed from these rabbits [41,121,122,123].

Domestic breeds of European rabbits were introduced to Australia from the time of the earliest European settlement in 1788, and although some local spread occurred it was the introduction of a small group of wild rabbits from Britain in 1859 that led to the continent-wide invasion and establishment of rabbits as Australia’s major vertebrate pest [124,125]. Myxomatosis was suggested as a potential biological control as early as 1918 [8,126] and trials were conducted during the 1930s to test lethality and species-specificity [51,52]. These were followed by field releases that failed to show potential for MYXV as a biological control because of limited dissemination in the rabbit population [5,8,127]. However, the initial field trials were conducted in very dry country where mosquitoes were absent, and further trials were subsequently conducted in higher rainfall zones.

Starting in December 1950, MYXV spread from one or more trial sites in the Murray Valley across vast swathes of south eastern Australia during the summer of 1950/51. This was facilitated by extensive inland flooding, allowing mosquito breeding in normally dry country, and the presence of huge numbers of completely susceptible rabbits at high population densities. It was also likely deliberately spread by landholders transporting infected rabbits, and there were other deliberate introductions outside the main epizootic but these seem not to have spread [3,4,8].

By the autumn, in March/April of 1951, the main epizootic was over both because mosquito activity declined with cooler weather and the profound loss of susceptible rabbits; however, the virus trickled on in isolated populations, usually ones not affected during the summer, and re-emerged in epizootic form in the following spring from September 1951 [3]. Natural spread of the virus in this second season was augmented by widespread inoculation campaigns and field studies were set up to monitor the outcomes of this accidental experiment. Introductions were also made to Tasmania and Western Australia, which were geographically separated from the initial epizootic [8,128].

The number of rabbits in Australia pre-1950 is not known but it was probably in the hundreds of millions and may have been an order of magnitude higher, as numbers had risen significantly in the immediate post-war period. Similarly, the reduction in population size during the first epizootic of 1950–1951 could not be measured, and only part of the continent was affected, but Ratcliffe *et al*. (1952) [3] described driving for days through formerly rabbit infested country and seeing only occasional survivors. They estimated that tens of millions of rabbits were killed, rather than hundreds of millions, but cautioned that they had little basis for the estimation. Localized population losses of 99%–100% were observed. Although rabbit numbers recovered in later years, the reduction in population was still marked: in the early 1990s it was estimated that the rabbit population was perhaps 5% of its previous level in the agricultural zone and perhaps 25% in the rangelands [129].

### 4.2. Attenuation of Field Strains of MYXV

The strain of MYXV released in Australia was termed the Standard laboratory strain (SLS), also called the Moses strain. The original inoculum was produced from Martin’s strain B (Martin 1936), which was obtained from the Rockefeller Institute in the USA and was originally derived from an infected European rabbit at the Institute Oswaldo Cruz in Brazil around 1910 [130]. The virus had been maintained by periodic passage in laboratory rabbits for over 40 years; case fatality rates (CFR) for laboratory and wild rabbits were estimated to be as high as 99.8% [8,127].

Once ecological studies were underway it became apparent that field strains of MYXV were emerging that were slightly attenuated compared to the progenitor SLS, and that they tended to outcompete SLS when both viruses were present [131,132,133]. By any conventional measure, these viruses were still highly virulent with CFRs of 90% or more but, on average, infected rabbits survived for longer in an infectious state than rabbits infected with SLS so the emerging field strains were more likely to be transmitted by mosquitoes.

While SLS was not a cloned virus in the sense of having been pock-purified on the chorioallantoic membrane of fertile eggs or cloned by limit dilution in rabbits, Fenner and Marshall (1957) [16] stated that there was no evidence that it was a mixture of virus populations based on repeatability of disease produced by inoculation of very low doses of virus. In addition, tests of 127 clones prepared from single pocks did not produce any prolonged survival times [8]. If this is correct, then attenuated field strains must have been initially derived by random mutations from SLS. This raises an interesting question of how the attenuated viruses arose, and were initially transmitted, particularly if this trait is polygenic. Attenuated viruses must be slightly less competitive, such that they will be selected against, in the rabbit in which the mutation first arose, and mosquito transmission will generally be associated with a substantial population bottleneck because only very low numbers of viral particles are delivered [55,134]. This implies that minor variants will usually be lost by chance.

The virulence of field isolates of MYXV was systematically analysed by infecting small groups of laboratory rabbits, usually five or six, and at least four months old, with low doses of each virus. Virulence was classified into five broad grades based on the CFR, average survival time (AST) and clinical signs [8,16]. The grade 3 classification was subsequently divided into 3A and 3B [135] (Table 5). The use of AST as a measure allowed the differentiation of very slightly attenuated strains that had CFRs of >99% but slightly longer survival times compared to SLS, while using low numbers of rabbits.

**Table 5:** Virulence grades of MYXV.

Virulence Grade	Case Fatality Rate (%)	Average Survival Time (Days) ^1^
1	99.5	≤13
2	95–99	14–16
3A	90–95	17–22
3B	70–90	23–29
4	50–70	29–50
5	<50	Not determined

^1^ Average survival time was calculated by transforming survival times using log_10_(ST-8) and then back-transformed. This was adjusted to allow for survivors either by the method of Sampford (1954) [136] or by allocating survivors a survival time of 60 days [16,135].

**Figure 1:**
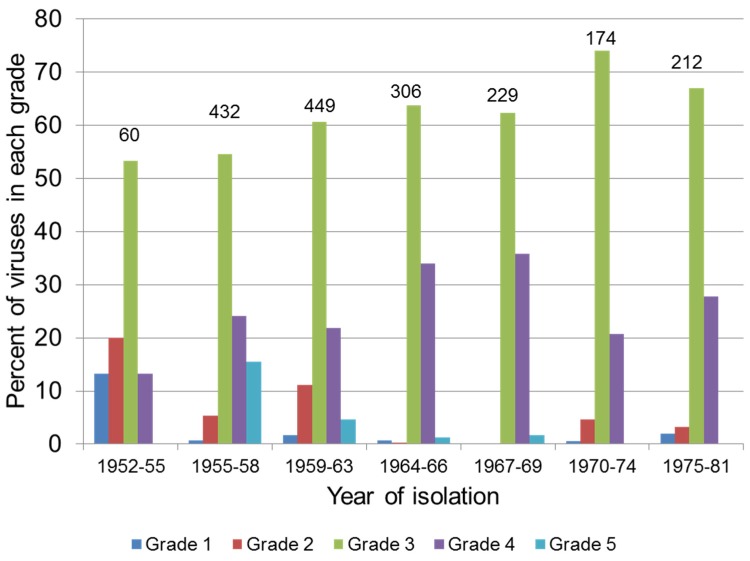
Virulence of Myxoma virus isolates in Australia 1952–1981. The proportion of MYXV isolates in each virulence grade (Table 5) is shown. Numbers above the bars indicate the number of isolates tested for each period. Grade 3A and 3B viruses are combined as grade 3. Data are from [137]. Figure is reprinted from Antiviral Research [64] (with permission from Elsevier).

Grade 1 viruses such as SLS quickly became relatively rare in the field and the predominant viruses were of grade 3 virulence with smaller numbers of grade 4 viruses (Figure 1). Where virulent viruses were isolated it was generally within a short time of release of SLS for biological control [16]. This situation persisted for the next 30 years [137]. While there has not been any systematic examination of field strain virulence since the 1980s, limited testing on small numbers of field strains from the 1990s showed that highly virulent grade 1 and attenuated grade 4 and 5 viruses were still present in the wild rabbit population [138,139].

**Figure 2:**
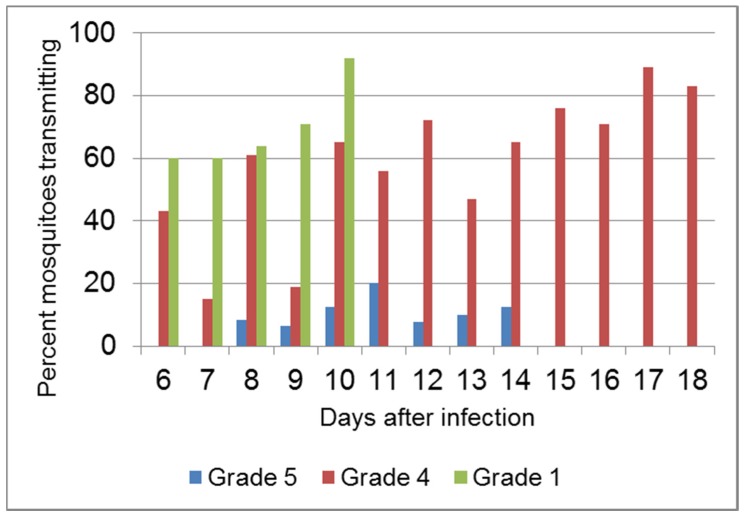
Mosquito transmissibility of Myxoma virus. The percentage of mosquitoes that successfully transmitted virus following feeding on cutaneous lesions of rabbits infected with grade 1, grade 4 or grade 5 viruses is shown. Data are from [134]. Figure is reprinted from Antiviral Research [64] (with permission from Elsevier).

MYXV can be transmitted by direct contact; virus is shed from eroded cutaneous lesions and mucosal sites such as conjunctivae and nasal passages and can be inoculated into the upper respiratory tract or conjunctivae during social interactions or cutaneously by fighting. However, for epizootic spread, mosquitoes or other mobile, biting arthropods are the most effective means of transmission. Mosquito transmission studies using laboratory rabbits infected with viruses of different virulence grades demonstrated that Grade 4 viruses were the most transmissible [134] (Figure 2). The titres of these viruses in the epidermis remained above the threshold for efficient mosquito transmission (10^7^ rabbit ID_50_/g) for long periods whereas grade 1 viruses, although rapidly reaching transmissible titres, killed the rabbits quite early. Grade 5 virus was poorly transmitted probably because the rabbit immune system was able to quickly control virus replication. The grade 5 virus, neuromyxoma, used in this study may not be representative of field strains as it reaches very low titres in the epidermis; other grade 5 viruses have higher titres but are still relatively quickly controlled in the epidermis [16,113].

### 4.3. Selection for Resistance in the Rabbit Population

Despite the transmission advantage of grade 4 viruses in laboratory rabbits, grade 3 viruses predominated in the field. The most likely explanation for this is that rabbits were undergoing intense selection for resistance to myxomatosis. The emergence of attenuated viruses allowed some survival of infected rabbits, although even in the first epizootic some rabbits recovered from infection [131,140]. Over time the breeding population would be dominated by rabbits that had survived myxomatosis potentially because of allelic variants that increased resistance. This selection may have been accelerated by high summer temperatures, which dramatically increase survival rates of rabbits infected with attenuated viruses especially rabbits with some genetic resistance [141,142].

In much of Australia, rabbits have an annual breeding season, during which does may produce 4–5 litters; the generation interval is approximately 12 months. Systematic studies using rabbits from a field site at Lake Urana in southern New South Wales demonstrated that over a seven year period, corresponding to seven annual epizootics of myxomatosis and approximately seven generations of selection, CFRs dropped from 90% to 26%. This was measured by infection of non-immune rabbits from each generation with the KM13 strain of MYXV under laboratory conditions [143,144].

Resistant rabbits have reduced disease severity and higher survival rates, rather than resistance to infection, and resistance can be overcome by viruses of higher virulence such as Cal MYXV or by suppressing the Th1 immune response [17,145,146]. The genetic basis of resistance is not known but it appears to be due to an enhanced innate immune response that controls virus replication in tissues distal to the inoculation site, effectively reducing the virulence grade of the infecting virus, and allowing development of an adaptive immune response that eventually clears the infection [113,114,147]. There is also the suggestion of a temporary enhanced resistance in rabbits born to does that had mated with males that had recovered from myxomatosis even if this male was not the sire of the kittens tested [148,149,150]. No experiments have been done to test these observations, which are based on post-hoc analyses of challenge data, and it is difficult to envisage a mechanism, perhaps epigenetic, by which this resistance might occur.

Although only limited studies were done, resistance does not seem to have emerged uniformly across Australia. It developed more rapidly in the hot dry areas, such as the Mallee in Victoria, than in cool moist climates [143,144,151] possibly due to less frequent epizootics in the cooler areas [57]. This means that selection pressure for transmission and hence virulence may have differed among climatic zones, and there is some evidence that more virulent viruses were more prevalent in the hotter dry regions where rabbits were generally more resistant [152], supporting the notion that transmission and virulence are strongly linked.

### 4.4. Virulence and Transmission

Modeling simulations suggest one MYXV strain should predominate: grade 4 in populations with no resistance but grade 3B in more resistant populations, which generally fits the observed field data [153,154]. However, there are obviously multiple virulence grades surviving in the field. This may occur because less common strains are only excluded over a long period [154]. Experimental releases of the virulent Lu strain [155,156,157,158] showed that field strains outcompeted the released virus suggesting that long-term coexistence of very virulent and moderately attenuated viruses is not likely at a local level. However, varying levels of genetic resistance and environmental patchiness may allow persistence of multiple virulence grades at some geographic scale [159]. Alternatively, viruses may switch between virulence grades relatively readily, and the most successful will depend on the immediate local conditions and resistance. Obviously, at a local scale, a highly lethal virus could exclude other viruses simply by being first to invade and eliminating the susceptible rabbits.

**Figure 3:**
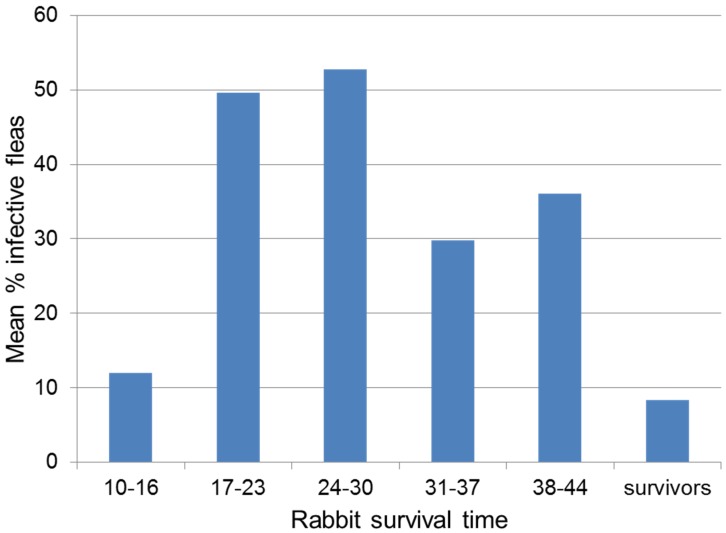
Transmission of MYXV by fleas 8-28 days after infection with viruses of different virulence grades. Fleas were combed from rabbits infected with viruses of virulence grade 1, 3A, 3B or 5, and tested for transmission by feeding on an uninfected rabbit. Rabbits have been grouped by survival time irrespective of the virus with which they were infected. The average percentage of infective fleas between days 8 and 28 after infection is shown for each group. Data are from [160].

An important nexus between virulence and transmissibility was further demonstrated by experiments with flea transmission of MYXV in wild rabbits. Rabbits that survived infection, regardless of the actual virus virulence, were relatively poor sources of virus based on the proportion of fleas that were able to transmit infection from the infected rabbit. The best transmission was from those rabbits that survived for several weeks with active myxomatosis but ultimately died (Figure 3). These were the rabbits infected with grade 3A viruses; survivors infected with the same viruses were poor sources of infectivity as, in general, were the rabbits infected with grade 1, grade 3B or grade 5 viruses [160].

Transmission is obviously a central component of virus fitness. The drivers of arthropod transmission are the duration of high titres of virus in the epidermis, whether the infected animal survives and the survival time. Importantly, the same virus will have a different disease phenotype (and transmission outcome) in resistant wild rabbits compared to unselected domestic rabbits, but even within wild rabbit populations individual levels of resistance will vary. In addition, biotic and abiotic factors that suppress the rabbit immune response, such as, age of the rabbit, nutritional stress, cold, parasites or intercurrent disease may lead to a more virulent phenotype and hence alter transmission potential [15,141,161,162,163]. Thus, under some circumstances, a highly attenuated virus could have quite successful transmission locally but be unable to invade a more resistant population [159].

## 5. MYXV Evolution in Europe

### 5.1. Introduction and Spread

In June 1952, the owner of an estate at Maillebois in north western France inoculated two wild rabbits with MYXV in an attempt to control rabbits on his property. The virus (Brazil/Campinas 1949) was obtained from the Virus Culture Collection in Lausanne, Switzerland and has since been known as the Lausanne strain (Lu). Unlike SLS, it had undergone very few passages in European rabbits since its isolation and infected rabbits had different lesion morphology to SLS with large purplish protuberant cutaneous lesions. However, the CFR and AST were indistinguishable from SLS in unselected rabbits [8,16]. As in Australia, rabbits in Europe were completely naïve to MYXV, despite earlier attempts at introduction [8,57], and the virus successfully established and gradually spread into the wild rabbit populations of Western Europe, Ireland and the United Kingdom [41,57]. Some dissemination was clearly deliberate, such as the introduction into Britain in 1953 [164]. However, unlike in Australia, strenuous efforts were made to control and disrupt the spread of MYXV in Europe but to no avail [40,41,57,127]. As well as spread into wild rabbits, MYXV also had significant impacts on the large rabbit farming industry, which produced domestic rabbits for meat and fur. In the UK it was estimated that the wild rabbit population fell by 99% following the establishment and spread of MYXV and for France estimates range from 90% to 95% [41,165].

Rabbits in Europe carried the European rabbit flea (*Spilopsyllus cuniculi*), which is an efficient vector of MYXV, and other fleas, with more limited geographic distribution, such as the Spanish rabbit flea (*Xenopsylla cunicularis*), were also efficient vectors. These fleas were not originally present in Australia but were later introduced to aid in the dissemination of MYXV [166,167,168]. Flea transmission of myxomatosis can occur throughout the year whereas mosquitoes and other flying vectors such as culicoides midges and simuliids (black files) are more seasonal. All of these vectors were important in different parts of Europe, although fleas were the main vector in Britain [41,164]. Critically, while the environment, climate, vectors and progenitor virus strain were all somewhat different to Australia, the evolutionary outcome was remarkably similar with emergence of attenuated viruses and selection for genetically resistant rabbits. However, there were differences in the rates at which attenuated viruses became dominant in the field and the emergence of resistance.

### 5.2. Attenuation in Europe

In France, the first attenuated virus was not isolated until April 1955, nearly three years after the initial introduction, whereas in Britain a grade 3 virus was isolated about 12 months after the initial outbreak [16]. Whether the difference in timing reflects lack of sampling, as occurred during the first epizootic in Australia, or different selection pressures is not clear. The intensive studies on virulence carried out by Fenner and co-workers in Australia were not replicated in Europe, although limited data from France shows that in 1962 the majority of viruses tested were of grade 3 virulence and in 1968 the majority were of grade 4 virulence [169]. This may reflect the slow emergence of resistance in wild rabbits in France (see below), or be biased by sampling from farmed rabbits that were not under selection for resistance [41]. In testing of small numbers of isolates from 1979 to 1982, grade 3 viruses again predominated [40]. More recent testing of 20 viruses, isolated from wild rabbits in Spain between 1992 and 1995, showed that the majority were of high virulence (equivalent to grade 1 or 2), although the testing protocol was different to previous studies [170].

**Figure 4:**
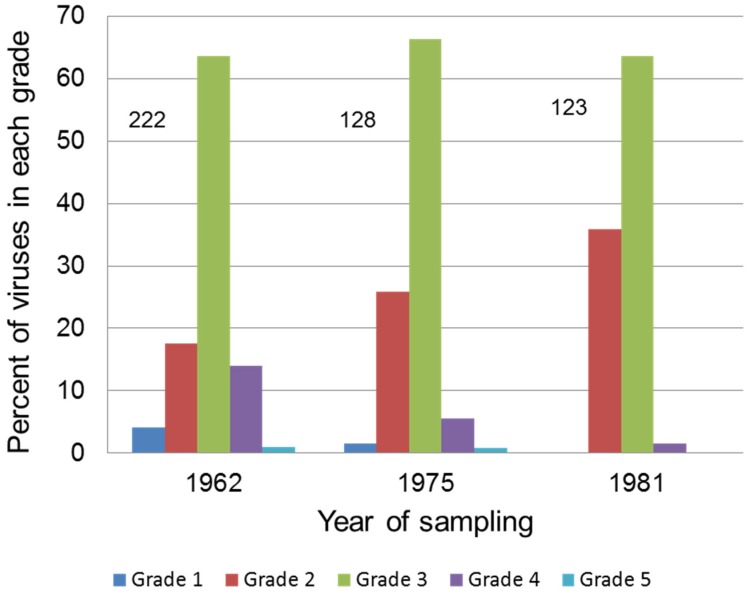
Virulence of Myxoma virus isolates in the United Kingdom. The proportion of MYXV isolates in each virulence grade for viruses isolated in the United Kingdom is shown. Numbers above the bars indicate the number of isolates tested. Data are from [171,172].

More systematic testing was done in Britain (Figure 4): in 1962, 64% of 222 viruses sampled were of grade 3 virulence and this predominance persisted in subsequent testing in 1975 (66%) and 1981 (63%). However, unlike in Australia, grade 2 viruses were always a substantial proportion of the isolates and grade 4 viruses were relatively rare especially after 1962. In 1981, nearly 36% of viruses tested were of grade 2 virulence, while grade 4 were less than 2%; this was accompanied by a marked reduction in grade 3B viruses and the disappearance of grade 5 viruses [171,172]. It was initially believed that the slower emergence of attenuated viruses was because fleas, the main vector in Britain, only left rabbits when the animal died and that this would lead to selection for more virulent viruses. However, it was subsequently shown that fleas were highly mobile and moved freely between live rabbits [173]. Moreover, it is clear that in parts of France and Spain mosquitoes and other flying insects were available as vectors [16].

### 5.3. Selection for Resistance in Europe

In France, resistance was quite slow to emerge in the wild rabbit population, although the systematic studies done in Australia were not replicated, no resistance was apparent in 1962 [8]. As in Australia, resistance appears to have been selected to different degrees in different regions, being reported as particularly strong in the Mediterranean south [40,41,57]. Similarly, in Britain, resistance was relatively slow to emerge; testing in 1966 showed that wild rabbits challenged with a grade 3 virus had longer survival times and a slightly higher survival rate compared to laboratory rabbits but the differences were relatively minor. However, by 1976, nearly 80% of rabbits from the same location survived challenge with the grade 3 virus (similar to the levels of resistance at Lake Urana in Australia after seven epizootics) and had prolonged survival times when infected with a grade 1 virus [174,175]. While resistance in Britain was widespread, the degree of resistance appeared to vary between geographic regions, as also occurred in France and Australia [175]. The emergence of strong resistance, possibly coupled with the dynamics of flea transmission, may be responsible for the increasing prevalence of grade 2 viruses in Britain.

### 5.4. Evolution of the Phenotype of Myxomatosis in Europe

Unlike in Australia, there was significant domestic rabbit breeding in Europe and the interchange of viruses between the wild and farmed populations may have had an impact on evolutionary dynamics of MYXV, especially when widespread but possibly inefficient vaccination was applied in farmed rabbits [176]. Arthur and Louzis (1988) [40] consider little interaction occurs between closed modern production units and wild rabbits but this would not have been the case for the large numbers of small holdings and backyard producers, especially during the early epizootics, and also for the producers of wild and cross-bred rabbits reared for release [41,162]. In domestic rabbit operations, destruction of infected rabbits to prevent transmission of myxomatosis would prevent selection for resistance [8], however, it could also inadvertently have selected for atypical clinical signs that were confused with bacterial respiratory infections and permitting ongoing transmission.

Two clinical types of myxomatosis have been described in France and Belgium, a dermatotrophic form characterized by multiple raised cutaneous tumours, typical of myxomatosis caused by Lu, and a “respiratory” or “amyxomatous” form in which the cutaneous tumours are not present, which appears to have emerged in the late 1970s [40,162]. However, the overall appearance of the affected rabbits can be very similar to those with the dermatrophic form, with swollen, closed eyelids, swollen heads and ears, mucopurulent rhinitis and blepharoconjunctivitis. The same clinical appearance is seen following either intradermal or intranasal inoculation and while it has been speculated that these “amyxomatous” biotypes of the virus have been selected for better transmission by contact, for example, via the upper respiratory tract or conjunctival inoculation, this has not been demonstrated [177]. Direct contact is needed for transmission as rabbits housed in adjacent cages did not transmit [162]. This amyxomatous form of disease has also been reported in wild rabbits [40,162]. It should, however, be recognized that myxomatosis has always been readily transmitted by direct contact [51,52].

Serological and virological evidence [178,179] indicates that many cases of myxomatosis in commercial rabbitries go unrecognized, implying that there may be ongoing transmission of attenuated viruses. Interestingly, some amyxomatous strains which caused severe disease in conventional farmed rabbits caused very mild clinical signs in laboratory rabbits maintained free of the common bacterial pathogens [118,177]. These observations suggest that MYXV strains that mimic, and may be dependent on, bacterial upper respiratory disease could be evolving in farmed rabbits.

It is likely that these unrecognized cases of myxomatosis are responsible for much of the speculation about how outbreaks of myxomatosis occur in apparently isolated populations. Reactivation and shedding of MYXV in recovered rabbits has been suggested to explain some outbreaks [162] and purportedly demonstrated [180]. However, subsequent attempts to demonstrate reactivation of virus were unsuccessful (unpublished data cited in [57]) [118]. Transfer of virulent virus between commercial farms, in vaccinated asymptomatic “carrier” rabbits, has been reported [181]. However, incubation periods of up to 20 days for some amyxomatous strains mean that rabbits could have been incubating the disease prior to vaccination [176]. In addition, vaccinated rabbits can become infected and shed MYXV following challenge [182].

Evolution of both Australian and European isolates of MYXV to produce a flatter less protuberant skin lesion has been described [8,135,171], although these lesions are still quite prominent compared to more recent Australian isolates. Some highly virulent modern field isolates of MYXV from Australian also produce a largely amyxomatous phenotype in laboratory rabbits with secondary cutaneous lesions being quite rare and the primary lesion at the site of inoculation extremely small and undifferentiated from the surrounding skin. However, these viruses tend to cause fairly typical myxomatosis in resistant wild rabbits [183].

### 5.5. Evolution of Vaccine Strains

Live virus vaccines have been extensively used in Europe to protect farmed and wild rabbits from myxomatosis [40,57,162,176,184,185]. Initially, only the heterologous RFV was available [14], but subsequently, a variant of the Cal MYXV MSD strain, attenuated by passage in cell culture, was used [186], and then local Lu-derived strains that were attenuated by cell passage were developed, such as the French SG33 [187]. Vaccination has led to some interesting evolutionary outcomes, for instance, the MSD vaccine strain appears to have established a transmission cycle in farmed rabbits [188]. In addition, the SG33 vaccine strain has undergone multiple recombination events with a Cal MYXV, presumably the vaccine strain [189,190]. A recombinant of MYXV with RFV has been described in the USA [191,192,193]; this probably occurred in a laboratory, and a recombinant between RFV and vaccinia virus has also been reported [194]. To date, there is no evidence of recombination between RFV and MYXV in the wild or farmed rabbit populations despite its earlier widespread use as a vaccine, including the use of RFV followed by boosting with SG33 in France.

## 6. Molecular Evolution of MYXV in Australia and Europe

**Figure 5:**
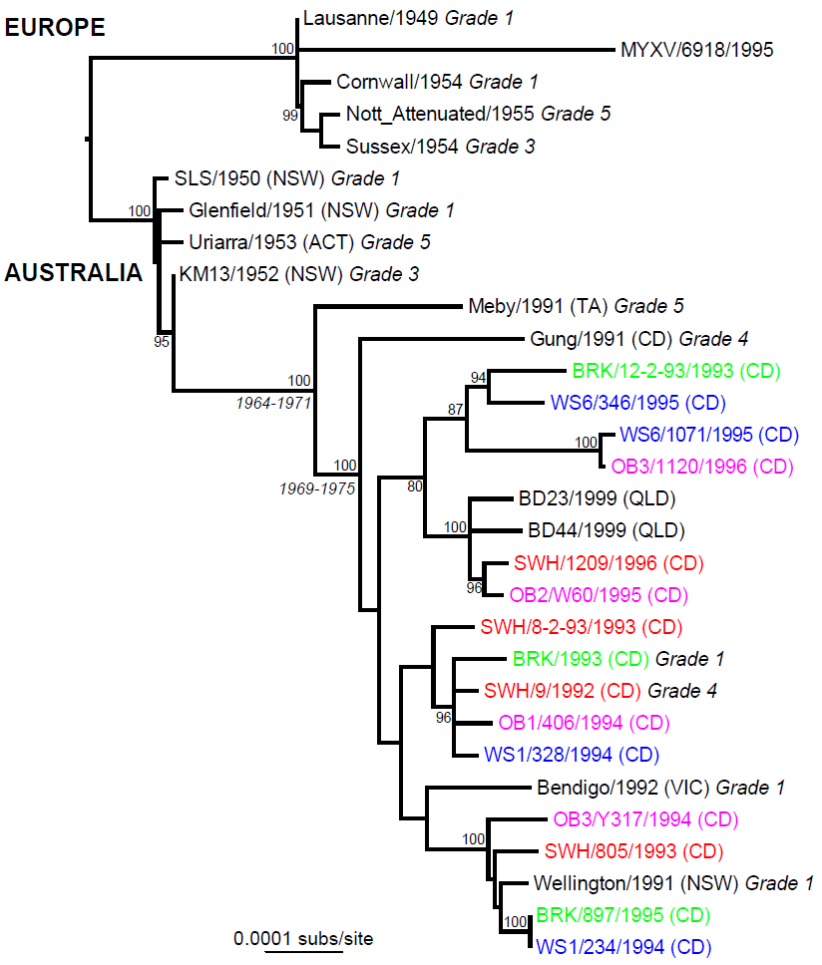
Phylogenetics and phylogeography of Myxoma virus in Australia and Europe. Samples are colour-coded according to location of sampling in the Canberra district of Australia (BRK, green; OB, pink; SWH, red; WS, blue; the state or region of sample collection is indicated in parentheses (ACT, Australian Capital Territory; CD, Canberra district; NSW, New South Wales; QLD, Queensland; TA, Tasmania; VIC, Victoria). Previously determined virulence grades are indicated as 1 to 5. Bootstrap values are shown for key nodes, and all horizontal branches are drawn according to the number of nucleotide substitutions per year. Credible intervals for divergence times for two key nodes are also shown (see [190,195] for details).

Genome sequencing studies from the Australian and European radiations show that MYXV has a very high evolutionary rate for a double strand DNA virus, at approximately 1 × 10^−5^ nucleotide substitutions per site per year, and that accumulate in a remarkably clock-like manner (Figure 5) [190,195]. This is similar to rates estimated for variola virus, the orthopoxvirus that caused smallpox in humans [196].

The progenitor Australian strain of MYXV, SLS, was isolated in Brazil nearly 40 years earlier than Lu and had been maintained by rabbit passage for 40 years. It has 72 nt differences from Lu including indels but only counting one TIR [190]. These include an indel in the 5' end of the *M005L*/*R* gene (a significant virulence determinant), an indel in the *M083L* gene and an indel in the 3' end of the *M152R* gene leading to read-through and an altered sequence at the C-terminus of the M152 protein. Whether these mutations were present in the original isolate or arose during rabbit passage cannot be determined. As already noted, while both SLS and Lu are of grade 1 virulence when tested in laboratory rabbits, Lu is actually more virulent than SLS when tested in resistant wild rabbits [137]. In addition, the two viruses have substantially different lesion morphology with Lu inducing large purple cutaneous tumours whereas SLS tends to induce a flatter (although still raised) skin lesion. Other South American isolates with limited rabbit passage history tend towards the Lu lesion morphology and the virus strain from which SLS was derived also tended towards a purple type lesion [16] suggesting that some mutations occurred during its passage history prior to release in Australia.

A rapid divergence occurred in Australia following the spread of MYXV in the period 1950–1953. Only two virus isolates from the first epizootic in the summer/autumn of 1950/51 were tested for virulence and both were grade 1 [16]. Genome sequencing of one, the Glenfield strain (Dubbo 2-51/1), isolated in February 1951, revealed multiple mutations including three single nucleotide indels that disrupt the *M014L*, *M130R* and *M153R* ORFs causing premature termination of translation. The M153 protein has a significant role in virulence by downregulating MHC-I from the surface of infected cells [106,107,108]. The function of M130 is not known but a gene knock-out virus was attenuated [91], although, Glenfield is more virulent than the progenitor SLS [197]. Interestingly, Glenfield has single non-conservative amino acid substitutions in three proteins involved in RNA transcription (M044: RNA helicase-nucleophosphohydrolase II; M108: DNA helicase-intermediate/late transcript release; M114: RNA polymerase subunit) that could potentially enhance virulence and which have not been seen in other sequences from Australia [190,195]. Although only six isolates were tested, by the end of the second epizootic in the autumn of 1952 (April and May), the viruses from a wide geographic area included slightly attenuated grade 2 and more attenuated grade 3 viruses but only two grade 1 viruses, although one of these showed prolonged survival in one rabbit [16]. Unfortunately, none of these viruses were available for sequencing.

Genome sequences have been completed for two viruses isolated during the third epizootic, the KM13 strain (Corowa 12-52/2), the prototype grade 3 virus, isolated in December 1952 [16,132], and the Uriarra (Ur) strain (Uriarra 2-53/1) isolated in February 1953 [198]. Ur was originally described as the prototype Australian grade 4 strain [16], but in all recent testing has been grade 5 virulence [113,199]. All three early isolates have the three indels found in SLS, confirming that these were definitely present in the progenitor virus; Ur and KM13 also share the indel in *M014L* found in Glenfield, suggesting that this mutation was relatively widespread. Ur has an additional indel in *M005L/R* at the same location as the original SLS indel plus an indel in the 3' end of the *M134R* gene, this is also present in KM13 and Glenfield but where these viruses have 3A inserted in M134, Ur only has 2A inserted leading to a predicted premature termination of translation. Both viruses have only low numbers of other substitutions but interestingly four amino acid substitutions in KM13 are present in all recent Australian isolates sequenced [190].

There are no Australian sequences for the period between 1953 and 1990. However, genome sequences for 21 viruses from 1991 to 1999 have been completed. Virulence classifications are known for six of these, with three viruses classified as grade 1 virulence and three of grade 4 or 5 virulence [138]. Molecular clock studies suggest that these 21 isolates share a common ancestor that was present between 1964 and 1971 [195]. Despite the high mutation rate, much of the genome is highly conserved: across all the Australian sequences 48 genes have no change and a further 23 have only synonymous substitutions. This small number of mutations in individual genes means any analysis of gene and site-specific selection pressure is highly problematic, in turn making it very difficult to determine which mutations have been responsible for initial attenuation and potentially reversion to virulence. There is no evidence for recombination in these data.

A common trend is the disruption of ORFs by single or multiple nucleotide indels, which frequently occurs in homopolymer sequences; in the Australian isolates 13 of 16 single nucleotide indels within coding sequences occur in homopolymer tracts (Table 6). The *M014L* ORF is disrupted in all three viruses sequenced from the early radiation. However, more recent sequences do not have the *M014L* ORF disrupted and intriguingly all of the modern viruses sequenced have an intact *M083L* ORF, despite this being disrupted in SLS and the early viruses. All but one of the recent viruses have the *M009L* ORF disrupted by an indel. Both the *M009L* indel and the *M083L* mutations occur on the long branch of the tree separating the 1951–1953 viruses from the 1990 viruses. Some virus isolates have further disruptions to the *M009L* ORF, or mutations forming stop codons, suggesting that it is becoming a pseudogene (Table 6).

**Table 6:** Indels disrupting coding regions of MYXV isolates from Australia and Britain [62].

Gene	Function (Protein Size aa)	Mutation	Effect	Virus (Virulence Grade if Determined)
*M000.5L/R*	Undetermined (72)	G del	Frameshift from aa 58 and readthrough stop codon adds 5 aa at C terminus	BD44
*M005L/R*	apoptosis inhibition/host range (483)	C insert (homopol)	Frameshift from aa 284 stop after aa 317; loss of C-terminal F box	WS6 346
*M005L/R*	apoptosis inhibition/host range (483)	C insert	Frameshift; stop after aa 73	Ur (5)
*M008.1L*	Secreted Serpin (369)	CC insert (homopol)	Stop after aa 299; loss of active site	BD44
*M009L*	BTB/kelch domains; putative Ub ligase (509)	A del (homopol)	Additional indel in disrupted ORF	SWH 8/2/93
*M009L*	BTB/kelch domains; putative Ub ligase (509)	TA insert	Additional indel in disrupted ORF	BRK (1)
*M009L*	BTB/kelch domains; putative Ub ligase (509)	A insert (homopol)	Early stop at aa 146—loss of all kelch domains	All recent Australian except Bendigo (1)
*M009L*	BTB/kelch domains; putative Ub ligase (509)	A del	Early stop at aa 114; additional indel in disrupted ORF	WS6 1071; OB31120
*M012L*	dUTPpyrophosphatase (148)	13 nt deletion	Stop after aa 70	OB3Y317
*M014L*	BTB/kelch domains; putative Ub ligase (517)	G insert (homopol)	Early stop at aa 477—loss of last kelch domain	Glenfield (1); KM13 (3); Ur (5)
*M018L*	cytoplasmic protein; VACV ^1^ F8 orthologue (66)	TT insert	Frameshift from aa 60; readthrough adds 20 aa at C-terminus	OB3Y317
*M036L*	VACV O1 orthologue (680)	92 nt del	Stop after aa 212	BRK (1)
*M036L*	VACV O1 orthologue (680)	T insert	Stop after aa 442	Sussex (3); Nottingham (5)
*M061R*	Thymidine kinase (178)	T insert (homopol)	Readthrough adds LKY to C-terminus	WS1 234
*M083L*	Carbonic anhydrase homologue/structural (286)	G insert (homopol)	Corrects G deletion in SLS; restores ORF	All recent Australian
*M130R*	Undetermined/virulence (122)	G insert (homopol)	Stop after aa 15	Glenfield (1)
*M134R*	Transmembrane protein (2000)	A insert (homopol)	Stop at aa 1953; retains predicted C-terminal transmembrane domain	Ur (5); Nottingham (5)
*M147R*	S/T protein kinase (288)	GT del (rpt seq)	Stop after aa 134	BD23
*M150R*	NF-κB inhibition (494)	TG insert	Stop after aa 196	Nottingham (5)
*M152R*	Serp 3 (266/273)	A del (homopol)	Stop after aa 271	WS6 1071; OB31120
*M153R*	Ub ligase/MHC-1 downregulation (206)	G del (homopol)	stop after aa 118	Glenfield (1)
*M153R*	Ub ligase/MHC-1 downregulation (206)	G insert (homopol)	Stop after aa 124	BD44
*M153R*	Ub ligase/MHC-1 downregulation (206)	T del	stop after aa 161	WS6 1071; OB31120
*M153R*	Ub ligase/MHC-1 downregulation (206)	73 nt del	Sequence read through replaces C-terminal CR domain of M153	Meby (5)
*M156R*	eIF2α homologue; IFN resistance (102)	T del	Read through stop—extra EG at C-terminus	WS6 346; OB3Y317

^1^ VACV—vaccinia virus.

Another group of viruses have deleted 923 nucleotides of the *M009L* gene, by what is essentially an expansion of the terminal inverted repeat boundaries, with duplication of 1635 nucleotides containing the *M156R*, *M154L* and part of *M153R* ORFs from the RH TIR boundary at the LH TIR. Whether this duplication of two potential immunomodulatory genes has an effect on viral fitness or virulence is not known. The *M009L* gene is itself a member of a three gene family (*M006L*/*R*, *M008L*/*R*, *M009L*), which may indicate an earlier duplication event during leporipoxvirus evolution. Fragmentation or duplication of genes has the potential to allow evolution of new functions, for example, the *CPXV12* gene in cowpox virus, is a gene fragment which has evolved a novel role in MHC-I downregulation and hence avoidance of T cell recognition [200].

Interestingly, *M009L* has also been lost by an expansion of the TIR boundaries and duplication of sequences from the RH end of the genome in the Cal MYXV MSW strain and, despite the disruption of at least two virulence genes, this virus is highly virulent in European rabbits.

The first reported outbreak of myxomatosis in Britain was in October 1953; sequencing of three viruses from the early radiation: Cornwall, April 1954 (grade 1); Sussex, September 1954 (grade 3); Nottingham, April 1955 (grade 5), showed that the virulent Cornwall had eight nucleotide differences from the Lu progenitor, seven of which were in ORFs and five were non-synonymous [190,195]. However, assuming that the British viruses were derived from a single introduction, divergent lineages had already arisen by 1954, since most of the mutations in Cornwall are not shared by the other two early viruses sequenced, and Sussex has mutations that are not present in Nottingham. Sussex has an indel disrupting the *M036L* ORF; this is also present in Nottingham, which has additional indels in the *M150R* gene and in the *M134R* gene. It seems likely that the multiple indels are responsible for attenuation of Nottingham, but whether the disruption to *M036L* is responsible for the attenuation of Sussex is not clear as a virulent Australian virus also has this gene disrupted [138]. Sussex shows that mutations associated with attenuation were present in the UK within less than 12 months of the initial outbreak, so that a lack of mutations was not responsible for the slower appearance of attenuated viruses in the UK. Hence these data tentatively suggest that there were different selection pressures operating in the UK compared to Australia.

The only other complete genome is that of the highly attenuated Spanish 6918 strain of MYXV isolated in 1995 [60]. Four genes in 6918 are disrupted by indels: *M009L*, *M036L*, *M135R* and *M148R*, the latter two have demonstrated roles in virulence [95,101], providing possible explanations for the extreme attenuation. The indel in *M036L* is independent of the mutation in Nottingham and the indel in *M009L* is different to that in the Australian isolates. Outside of these indels there were 67 nucleotide substitutions between the grade 5 6918 strain and the Lu progenitor over the 43 years of evolution in European rabbits. Viruses with the same mutation in the *M135R* gene as 6918 have been isolated in the Netherlands and so may be widely distributed in Europe [201] but, in Australian viruses, no mutations have been found in this gene [190]. Interestingly, RFV has lost the *M135* gene orthologue [61]. In partial sequence studies, various Spanish MYXV isolates also had disruptions to *M009L* and *M036L* as well as *M002L/R* and *M017L* [202] and some Portuguese isolates had *M009L* disrupted [203].

The poxvirus early, intermediate and late promoter sequences appear to be conserved across the leporipoxviruses sequenced [61]. However, the impact of level of gene expression on fitness and virulence is not clear. While selection for alteration in gene expression is an attractive concept for modifying virulence, the fact that in many cases promoter sequences are embedded within the 3' end of the preceding gene may tend to conserve these sequences. Examination of potential promoter sequences across all of the MYXV genomes sequenced revealed only six mutations and most of these would be predicted to only have minimal effects based on previous mutational analysis of the vaccinia virus promoter sequences [190,204,205].

Overall, the conclusion from the genome studies is that in these large, complex DNA viruses there is no common pathway to attenuation or virulence, but a convergence for phenotype that is compatible with multiple different genotypes and possibly involving complex epistatic interactions. Variants would undergo local selection but, as already noted, it is likely that a broad range of genotypes is compatible with successful spread depending on the local ecological conditions and rabbit population dynamics, including the level of resistance in the population. However, it is clear that from very early in the adaptation of MYXV to European rabbits, these local variants outcompeted virulent viruses released into the same populations [4,133,155]. The dynamics of selection must be altered in resistant rabbit populations potentially driving selection for more virulent viruses, when measured in laboratory rabbits.

The sequence data for RFV and Cal MYXV suggest that in some evolutionary pathways loss of genes is compatible with successful transmission, but whether these genes were actively selected against to enhance transmission by reducing virulence cannot be determined. Similar reductive evolution potentially associated with adaptation to new environments or selection for attenuation has been described in the orthopoxviruses [206,207]. The relatively large number of mutations seen in 50 years of evolution of MYXV in European rabbits potentially provides many pathways by which changes in virulence may occur and, as has occurred in MSW and RFV, it may be that a number of mutations are compatible with increased fitness and that which ones get fixed in the population in the new host species may in part reflect stochastic factors such as local extinction of host populations as much as active selection.

## 7. Phylogeography

Geographically, multiple lineages of MYXV can coexist over quite small distances with particular variants dominant on sites in different years and multiple variants isolated on single study sites during a season [139,190,195] (Figure 5). At face value, this suggests that there is little difference in overall fitness of these viruses at a local scale, but that regionally successful variants arise and are lost depending on the geographic scale under consideration and rabbit population dynamics [190]. However, phylogenetically closely related viruses have been isolated over 1000 km apart in the dry hot region of SW Queensland and in the cooler, higher rainfall, Canberra district suggesting that widespread dispersion of viruses can occur. Although the mechanism of spread over such distances is unclear there is some evidence for long distance dispersal via mosquitoes, for example, to isolated islands [3,8].

The emergence and gradual domination of attenuated strains of MYXV in Australia occurred in the face of ongoing widespread releases of the virulent SLS and, in NSW and later Victoria, the SLS-derived Glenfield strain. Following its spread in Europe, the Lu strain was released experimentally in Australia in 1954 [155] but was outcompeted by local field strains. Despite this early failure, Lu was widely released for rabbit control in Australia from the 1970s to the 1990s in conjunction with the establishment of the European rabbit flea as an additional vector. However, there is no evidence from either genomic sequencing or from restriction fragment length polymorphism analysis of hundreds of samples that Lu has established in Australia and contributed to the existing field strains [138,139,158,183,190]. Phylogenetically, all the recently sampled viruses are distinct from SLS, Glenfield and Lu suggesting that the ongoing releases of these viruses made no significant contribution to the subsequent evolution of myxoma virus in Australia.

## 8. Future Evolution of Host and Pathogen

Sequence studies provide the basis for understanding the molecular changes that led to attenuation, although it is difficult to determine the roles of individual mutations and gene disruptions. Similarly, it is difficult to know whether virulence was maintained by circulating virulent viruses or re-evolved in attenuated viruses. That is, do particular virulence lineages maintain stable circulation or is it relatively easy to flip back and forth between virulent and attenuated viruses with local selection determining which phenotype is successful? Further important questions include whether there is ongoing selection pressure on the virus driven by increasing resistance in the rabbit population, what capacity the rabbit has for further evolution, and what the cost of resistance is to the rabbit? This is further complicated by ongoing selection pressure on the European rabbit imposed by the virulent rabbit haemorrhagic disease virus (RHDV), a calicivirus, first described in China in 1984, that has spread in Europe and, more recently, Australia causing high mortality in rabbit populations [208,209].

Writing 50 years ago, Fenner and Ratcliffe (1965) [8] suggested two possible evolutionary trajectories. First, that MYXV and the European rabbit evolve towards a fibroma type of disease as is seen in *S. brasiliensis* or *S. bachmani*.

Such an outcome is dependent on the rabbit being able to evolve very strong resistance that prevents dissemination and generalized disease, limiting virus replication to the inoculation site. This would require the virus to maintain sufficient immune suppression capacity to prevent clearance at the inoculation site but not overwhelm the rabbit. Obviously, the previously characterized grade 5 viruses would not be selected as they would be too readily controlled by the rabbit immune system. Viruses selected in these conditions of high resistance could be of very high lethality for non-resistant rabbits. The alternative is that the virus mutates to lose the ability to disseminate within the host while retaining its resistance to immune clearance from the inoculation site (perhaps by recombination with RFV). However, it is questionable how well such a virus would compete with field strains that cause generalized disease, because multiple virus-rich tissues would provide more opportunities for insect transmission than a single fibroma unless it had very prolonged persistence.

The second possibility was that selection for genetic resistance in the rabbit population reaches a plateau because further selection for virus virulence is not possible.

In this scenario, a relatively stable situation occurs whereby virus strains with sufficient virulence to cause the generalized lethal disease optimal for transmission continue to predominate in the field, causing appreciable mortality in the population, but not driving further selection. Rabbit resistance may also plateau because the costs of resistance are too high or there is insufficient genetic polymorphism for further selection in the absence of mutation in the rabbit genome. Depending on the eventual level of resistance in the rabbit population, this could see the emergence of viruses with hypervirulent phenotypes in unselected rabbits.

A third option not considered by Fenner and Ratcliffe (1965) [8] is that subtypes of the virus could evolve towards contact transmission as may be occurring in Europe with the evolution of amyxomatous strains of virus. On rabbit farms it is possible that simultaneous coinfection with bacteria causing respiratory disease could mask the clinical signs of myxomatosis and by suppressing the rabbit’s immune response help to maintain the transmission of very attenuated viruses. Such transmission is likely only to be sustainable where high rabbit densities and close contact prevail.

After 65 years of evolution, there is very little evidence for the emergence of a fibroma type disease and some evidence that grade 1 and 2 viruses are more common in the field in Europe and Australia, suggesting that genetic resistance may still be driving virus evolution. Clearly, further systematic studies of viruses and rabbits are needed to understand how this complex evolutionary story will play out and to understand the biological and molecular mechanisms of this evolution. A single model may not prevail with different environments favouring different evolutionary solutions.
